# Targeted PET Imaging of *Pseudomonas aeruginosa* Using a [^68^Ga]Ga-Pyoverdines Cocktail

**DOI:** 10.2967/jnumed.125.271909

**Published:** 2026-11

**Authors:** Kristyna Krasulova, Barbora Neuzilova, Katerina Dvorakova Bendova, Katarina Hajduova, Patrik Mlynarcik, Miroslav Popper, Marian Hajduch, Milos Petrik

**Affiliations:** 1 Faculty of Medicine and Dentistry, Institute of Molecular and Translational Medicine, Palacky University, Olomouc, Czech Republic;; 2 Department of Microbiology, Faculty of Medicine and Dentistry, Palacky University Olomouc, Olomouc, Czech Republic;; 3 Czech Advanced Technology and Research Institute, Palacky University, Olomouc, Czech Republic; and; 4 Institute of Molecular and Translational Medicine, University Hospital, Olomouc, Czech Republic

**Keywords:** radiopharmaceuticals, [^68^Ga]Ga-pyoverdines, PET, *Pseudomonas aeruginosa*, imaging, infection

## Abstract

*Pseudomonas aeruginosa* is a major cause of nosocomial infections. Current diagnostic methods often lack sensitivity and specificity, resulting in a delayed or inaccurate diagnosis. To address these limitations, new diagnostic strategies are being developed. Molecular imaging techniques, such as PET, allow localization and monitoring of infections in situ; however, specific radiotracers for *P. aeruginosa* imaging are lacking. Radiolabeled siderophores have shown promise as diagnostic tools for PET imaging. Pyoverdines, specific siderophores produced by *P. aeruginosa*, are classified into 3 types: pyoverdine type I (PVDI), pyoverdine type II (PVDII), and pyoverdine type III (PVDIII). In this study, we evaluated a radiotracer specifically targeting *P. aeruginosa* infections using radiolabeled pyoverdines. **Methods:** We radiolabeled PVDI, PVDII, and PVDIII with ^68^Ga and evaluated their stability, in vitro uptake by microbial cultures, and in vivo biodistribution in healthy animals and 2 animal infection models. A cocktail of [^68^Ga]Ga-PVDI, [^68^Ga]Ga-PVDII, and [^68^Ga]Ga-PVDIII was used for PET imaging. **Results:** The radiolabeled pyoverdines exhibited high in vitro and in vivo stability, rapid renal clearance, and low blood retention. In vitro uptake using reference strains and clinical isolates showed that each strain had high uptake of one specific pyoverdine type. The cocktail successfully detected all tested *P. aeruginosa* strains in vivo, with no tracer accumulation observed in sterile inflammation, other pathogens, or heat-inactivated bacterial cells, confirming its specificity. **Conclusion:** A [^68^Ga]Ga-pyoverdines cocktail was developed as a specific PET imaging agent for detecting *P. aeruginosa* infections. Combining 3 radiotracers for broad strain coverage represents a significant advance in the accurate diagnosis of *P. aeruginosa* infections and confirms the potential utility of radiolabeled siderophores in PET imaging of infections.

## Visual Abstract

**Figure:**
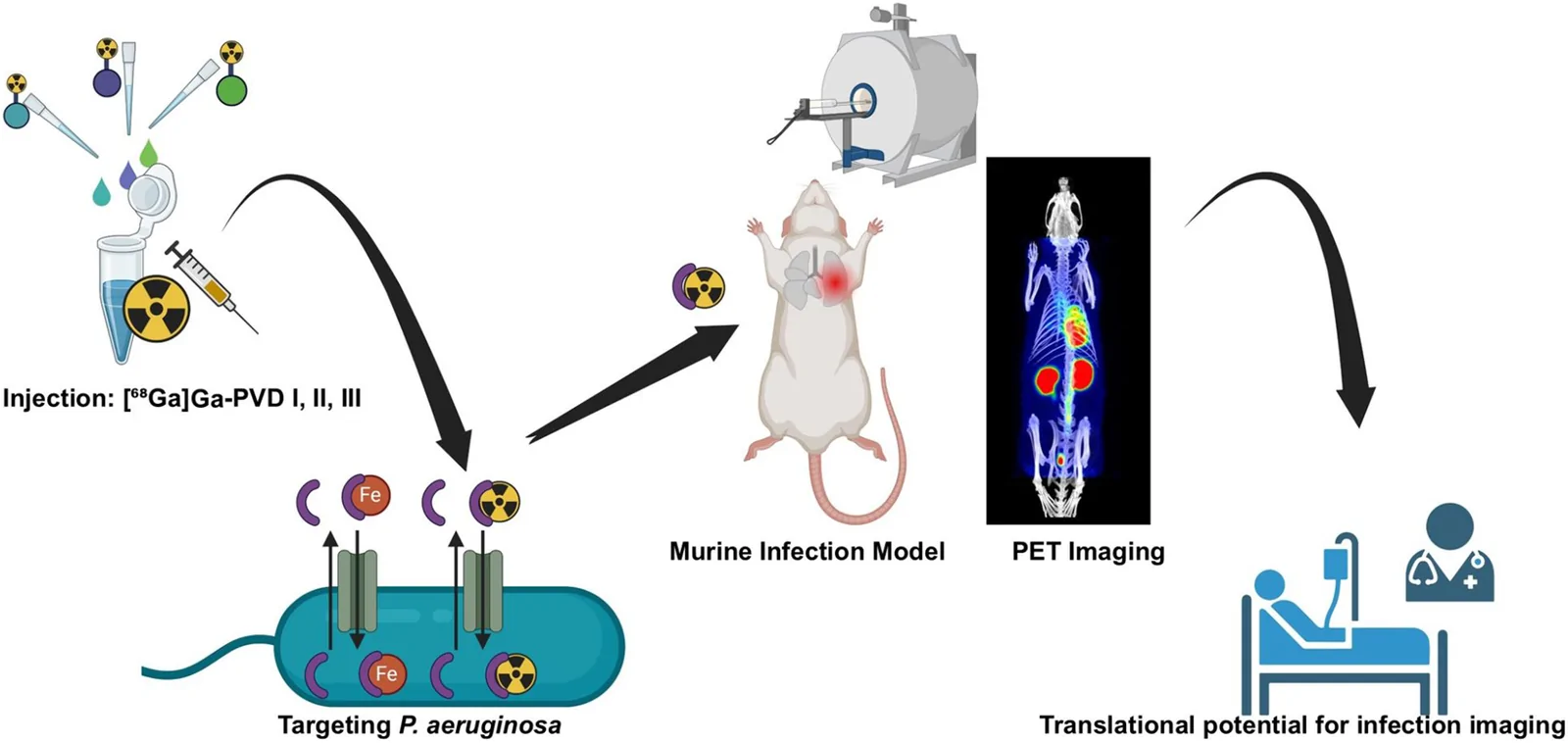

Infections are a major global health challenge, ranking third in mortality and first in morbidity among all human diseases (1,2). Current diagnostic methods, including microbiologic culture and molecular techniques, rely on clinical samples (e.g., blood, urine, stool, cerebrospinal fluid). Preliminary diagnostic results can often be obtained within 24 h (3). However, these samples may not accurately represent the microbial environment at the infection site, leading to missed or incomplete diagnoses. Sampling limitations can fail to capture infection heterogeneity, such as multiple lesions or temporal changes during disease progression and treatment. To address this, significant efforts are underway to develop innovative therapeutic and diagnostic strategies. Among them, molecular imaging techniques such as SPECT and PET are gaining prominence. These methods enable in situ infection localization via radiopharmaceutical uptake and can be combined with anatomic imaging modalities, such as CT and MRI, forming hybrid techniques that enhance diagnostic accuracy (4). Currently available radiopharmaceuticals lack specificity for infectious tissue, as they often cannot distinguish infection from inflammation or malignancy. One promising strategy for pathogen detection and localization is the use of radiolabeled siderophores (5,6).

Siderophores are small, iron-chelating molecules produced by almost all microorganisms and plants. These compounds are pathogen-specific and do not interact with the host system. They are designed to scavenge and transport iron from the environment and have a specific binding site for iron (7). ^68^Ga, a positron-emitting radionuclide with ironlike properties, can easily replace iron in siderophores to form radiolabeled complexes that have shown promise as PET tracers for accurate imaging of infections (8,9).

*Pseudomonas aeruginosa* is a leading cause of nosocomial infections, particularly in immunocompromised and critically ill patients, as well as those with cystic fibrosis. The prevalence of *P. aeruginosa* poses a significant challenge to clinicians because of its ability to become multidrug-resistant and its high mortality rate (10). *P. aeruginosa* produces a group of siderophores called pyoverdines. These siderophores play a crucial role in bacterial physiology, contributing to processes such as virulence and the formation of biofilms. The diversity of pyoverdines among *Pseudomonas* species is significant (11). Meyer et al. (12) analyzed pyoverdines and classified them into 3 different types. Pyoverdine type I (PVDI) is identical to *P. aeruginosa* ATCC15692, type II (PVDII) is identical to *P. aeruginosa* ATCC27853, and type III (PVDIII) is identical to *P. aeruginosa* DSM3602 (Fig. 1). [^68^Ga]Ga-PVDI has already been introduced by Petrik et al. (13) for specific imaging of *Pseudomonas* infection using PET. However, that study focused on a single strain of *P. aeruginosa*.

**FIGURE 1.**
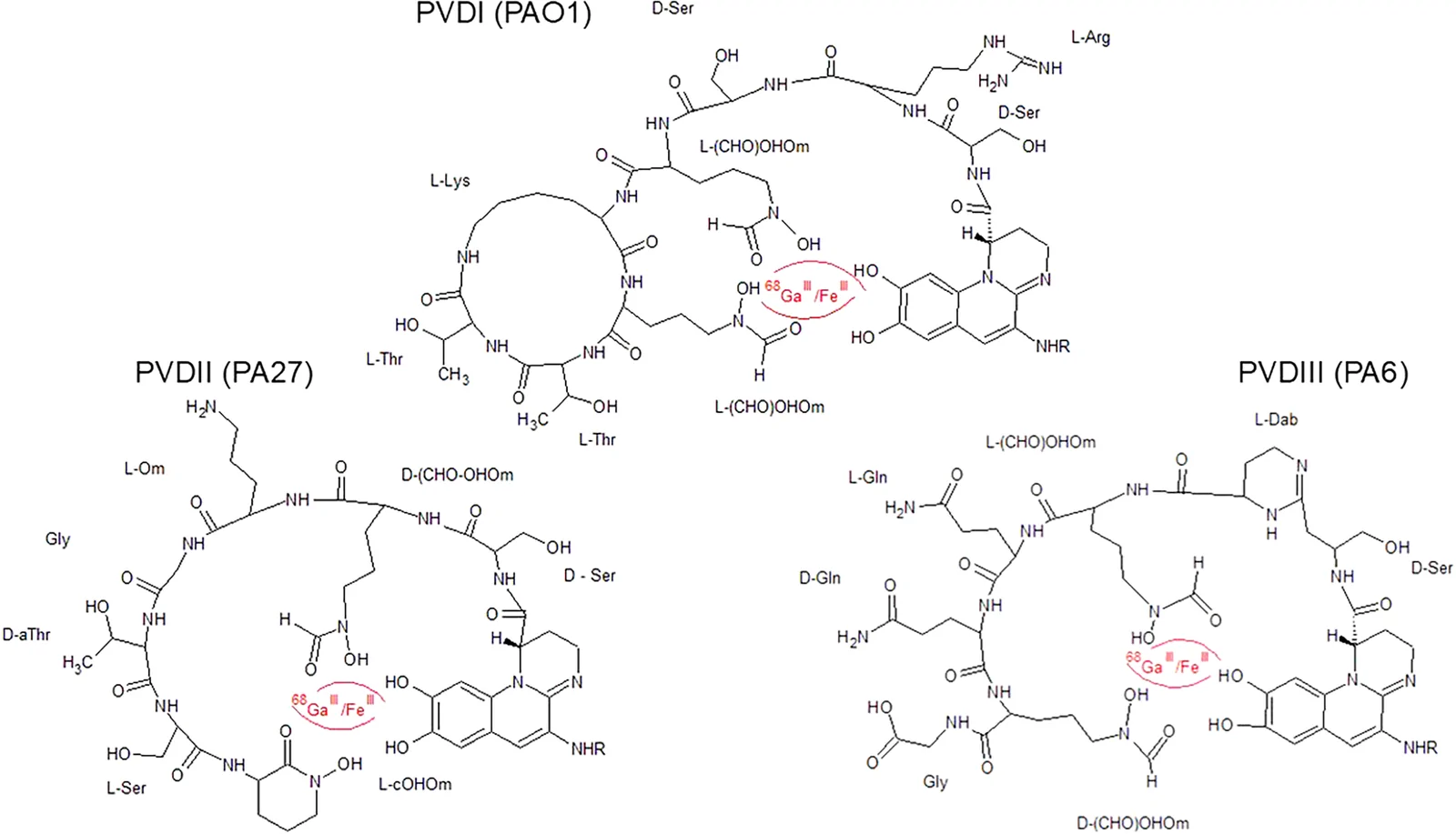
Chemical structures of 3 Fe^III^/^68^Ga^III^-pyoverdines, adapted from (12).

Here, we describe the use of a ^68^Ga-labeled pyoverdine cocktail for identifying a broad range of *P. aeruginosa* strains and evaluate its stability, in vitro uptake by microbial cultures, and in vivo biodistribution in healthy animals and 2 animal infection models.

## MATERIALS AND METHODS

### Animal Experiments

Female BALB/c mice and Lewis rats age 8–10 wk (Envigo) were used in the experiments. All injections, bacterial infection, and imaging procedures were performed under 2% isoflurane anesthesia (FORANE; Abbott Laboratories) to reduce suffering and prevent animal movement.

### PET Imaging

Animals were injected with 5−10 MBq of [^68^Ga]Ga-PVDI, [^68^Ga]Ga-PVDII, [^68^Ga]Ga-PVDIII or [^68^Ga]Ga-pyoverdines cocktail (via the tail vein) and placed in the prone position in a NanoScan PET/CT or NanoScan PET/MRI 3T system (Mediso Medical Imaging Systems). After the administration of [^68^Ga]Ga-labeled tracers, static PET imaging was initiated at 45 min postinjection, followed by whole-body helical CT or MRI scans. The final images were normalized to the injected activity and animal weight, with the results presented on an SUV scale.

A full version of the materials and methods is provided in the supplemental materials, available at http://jnm.snmjournals.org.

## RESULTS

### Radiolabeling

PVDI, PVDII and PVDIII were radiolabeled with ^68^Ga with high radiochemical purity (>95%) confirmed by thin-layer and high-performance liquid chromatography (Supplemental Fig. 1). Pyoverdines were radiolabeled with ^68^Ga with molar activities of up to 5 GBq/µmol for PVDI and 8 GBq/µmol for PVDII and PVDIII. [^68^Ga]Ga-pyoverdines were stable within 2 h in human serum, diethylenetriaminepentaacetic acid, and phosphate-buffered saline. All [^68^Ga]Ga-pyoverdines were highly unstable in media with a high iron concentration. The radiocomplexes also exhibited hydrophilic properties and low protein binding. The in vitro characteristics of [^68^Ga]Ga-pyoverdines are summarized in Table 1.

**TABLE 1.** In Vitro Characterization of [^68^Ga]Ga-Pyoverdines (*n* = 3)

Incubation time	Protein binding (%)	Stability in human serum (%)	Stability in 0.1 M FeCl_3_ (%)	Stability in 6 mM DTPA (%)	Stability in PBS (%)
[^68^Ga]Ga-PVDI					
30 min	2.31	97.90 ± 0.52	0.53 ± 0.32	94.80 ± 2.21	97.43 ± 1.36
60 min	2.79	96.60 ± 1.19	0.37 ± 0.21	95.70 ± 0.62	95.94 ± 1.57
120 min	2.46	95.60 ± 2.34	0.17 ± 0.09	89.70 ± 2.73	95.23 ± 1.01
[^68^Ga]Ga-PVDII					
30 min	0.93	94.03 ± 4.86	1.13 ± 0.90	96.30 ± 2.26	95.73 ± 1.10
60 min	0.88	94.53 ± 4.23	0.67 ± 0.55	97.40 ± 1.50	93.70 ± 1.41
120 min	0.66	93.17 ± 5.16	0.20 ± 0.14	94.53 ± 3.67	97.23 ± 0.45
[^68^Ga]Ga-PVDIII					
30 min	2.82	95.30 ± 2.11	1.30 ± 1.15	95.20 ± 0.26	97.00 ± 0.11
60 min	3.19	96.77 ± 1.07	1.17 ± 0.92	96.40 ± 0.84	95.70 ± 1.57
120 min	3.94	94.63 ± 2.41	0.35 ± 0.35	96.85 ± 0.21	97.10 ± 0.50

Log P values were 3.07 ± 0.08 for [^68^Ga]Ga-PVDI, −3.46 ± 0.06 for [^68^Ga]Ga-PVDII, and −3.17 ± 0.19 for [^68^Ga]Ga-PVDIII.

DTPA = diethylenetriaminepentaacetic acid.

Data for [^68^Ga]Ga-PVDI were previously published in (13).

### In Vitro Uptake Assays

Each tested reference strain demonstrated a high uptake of only one pyoverdine type. Specifically, 4 strains showed high uptake of [^68^Ga]Ga-PVDI, 6 of [^68^Ga]Ga-PVDII, and 1 of [^68^Ga]Ga-PVDIII (Fig. 2A). Patient-isolated *P. aeruginosa* strains were also tested, with a high uptake of only one pyoverdine type: 1 strain showed high uptake of [^68^Ga]Ga-PVDI, 6 of [^68^Ga]Ga-PVDII, and 3 of [^68^Ga]Ga-PVDIII. However, the uptake rates of patients’ isolates were lower than those of the reference strains (Fig. 2B). The observed distribution of pyoverdine uptake confirmed the siderotyping of *P. aeruginosa* strains into 3 distinct groups (Fig. 2). Supplemental Table 1 provides the list of microbial strains used in the study.

**FIGURE 2.**
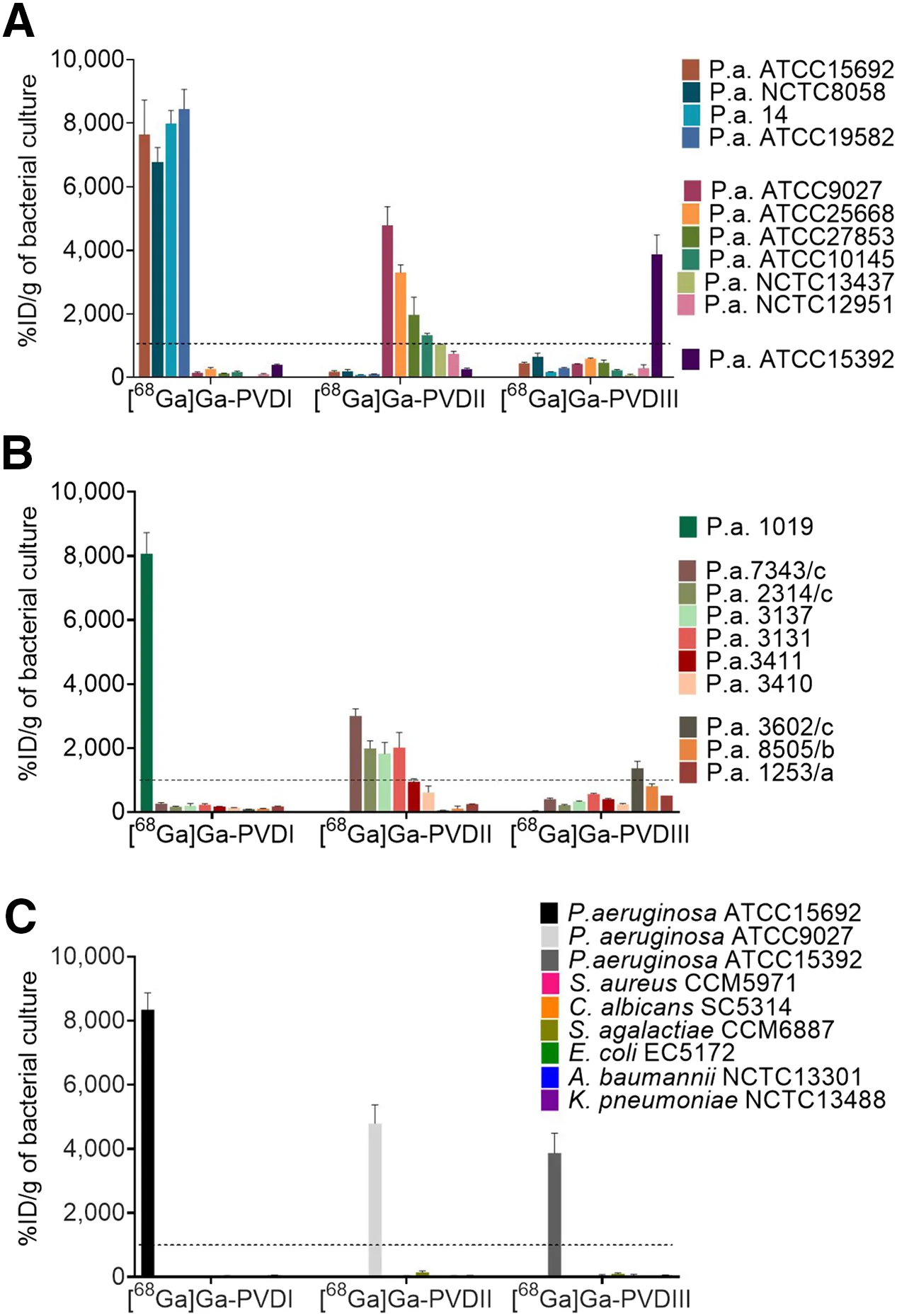
In vitro uptake of [^68^Ga]Ga-pyoverdines in different *P. aeruginosa* (P.a.) reference strains (A), patient isolates (B), and other selected pathogens (C) after 45 min of incubation in Mueller–Hinton medium. High uptake limit is represented by dashed line.

Preincubation with an excess of Fe-desferrioxamine B (DFO-B), a competitive siderophore complex, significantly reduced the uptake of radiolabeled pyoverdines compared with cultures not preincubated with DFO-B. Furthermore, uptake was completely abolished when microbial cultures were heat-inactivated at 95 °C for 40 min (Supplemental Fig. 2). None of the other pathogens tested demonstrated uptake of any [^68^Ga]Ga-pyoverdines (Fig. 2C).

### In Vivo Stability

[^68^Ga]Ga-pyoverdines demonstrated satisfactory stability in vivo at 30 and 90 min postinjection. In urine, stability at 30 min postinjection remained above 92% for all [^68^Ga]Ga-pyoverdines; at 90 min postinjection, stability decreased to 74% for [^68^Ga]Ga-PVDI, 86% for [^68^Ga]Ga-PVDII, and 59% for [^68^Ga]Ga-PVDIII. In blood, stability was measured at 5 min postinjection, with [^68^Ga]Ga-PVDI showing the highest stability (93%), followed by [^68^Ga]Ga-PVDIII (84%) and [^68^Ga]Ga-PVDII (74%). Detailed in vivo stability results of [^68^Ga]Ga-pyoverdines are summarized in Table 2.

**TABLE 2.** In Vivo Stability of [^68^Ga]Ga-Pyoverdines in Murine Urine and Blood

Incubation time	Stability in urine (%)	Stability in blood (%)
[^68^Ga]Ga-PVDI		
0 min	97.55 ± 0.21	
5 min	–	93.20 ± 0.84
30 min	92.50 ± 0.28	
90 min	74.55 ± 3.32	
[^68^Ga]Ga-PVDII		
0 min	95.95 ± 0.91	
5 min	–	73.65 ± 3.74
30 min	92.90 ± 0.56	
90 min	86.50 ± 0.42	
[^68^Ga]Ga-PVDIII		
0 min	98.10 ± 0.42	
5 min	–	83.85 ± 1.48
30 min	92.90 ± 0.57	
90 min	58.85 ± 3.04	

### Ex Vivo Biodistribution

Biodistribution studies in BALB/c mice demonstrated low blood-pool retention and rapid renal clearance for all [^68^Ga]Ga-pyoverdines. Blood pool retention decreased from 30 to 90 min for [^68^Ga]Ga-PVDI (0.81 ± 0.04 %ID/g to 0.09 ± 0.01 %ID/g), [^68^Ga]Ga-PVDII (1.12 ± 0.29 %ID/g to 0.06 ± 0.00 %ID/g), and [^68^Ga]Ga-PVDIII (0.98 ± 0.02 %ID/g to 0.04 ± 0.00 %ID/g). At 90 min, mean renal uptake was 1.95 ± 0.43 %ID/g for [^68^Ga]Ga-PVDI, 2.19 ± 0.20 %ID/g for [^68^Ga]Ga-PVDII, and 1.05 ± 0.03 %ID/g for [^68^Ga]Ga-PVDIII. Rapid clearance and no retention were observed for other monitored organs. The ex vivo biodistribution data for [^68^Ga]Ga-pyoverdines are summarized in Supplemental Figure 3.

### Animal Imaging Studies

#### [^68^Ga]Ga-Pyoverdines Imaging

PET/CT imaging of healthy mice injected with [^68^Ga]Ga-PVDII and [^68^Ga]Ga-PVDIII confirmed the data from the ex vivo biodistribution studies, as well as the previously published data for [^68^Ga]Ga-PVDI (13). All [^68^Ga]Ga-pyoverdines were rapidly cleared from the bloodstream via renal excretion and showed no excessive accumulation in other organs (Supplemental Fig. 4).

The strain-specific in vitro uptake of [^68^Ga]Ga-PVDI, [^68^Ga]Ga-PVDII, and [^68^Ga]Ga-PVDIII was confirmed by in vivo imaging studies in infected mice, demonstrating their specificity for imaging *P. aeruginosa* infections. Accumulation of [^68^Ga]Ga-PVDI signal was observed at the site of *P. aeruginosa* ATCC15692 injection, with negligible or low signal at sites infected by strains ATCC9027 and ATCC15392. Similarly, [^68^Ga]Ga-PVDII showed significant accumulation at sites infected by *P. aeruginosa* ATCC9027, whereas no signal was observed for the other strains. Finally, [^68^Ga]Ga-PVDIII showed significant accumulation at the site of the infection caused by *P. aeruginosa* ATCC15392, with a negligible signal for strain ATCC15692 and no signal for strain ATCC9027 (Fig. 3; Supplemental Fig. 5). No radioactive signal was detected in the right hind legs, which were injected with saline and served as a negative control. The findings were confirmed by statistical analysis of the quantification results for all [^68^Ga]Ga-pyoverdines (Fig. 3; Supplemental Fig. 5). When clinical isolates of *P. aeruginosa* were used, PET signals from individual [^68^Ga]Ga-pyoverdines were positive only for the corresponding strain (Supplemental Fig. 6). These results are in full agreement with the in vitro experiments.

**FIGURE 3.**
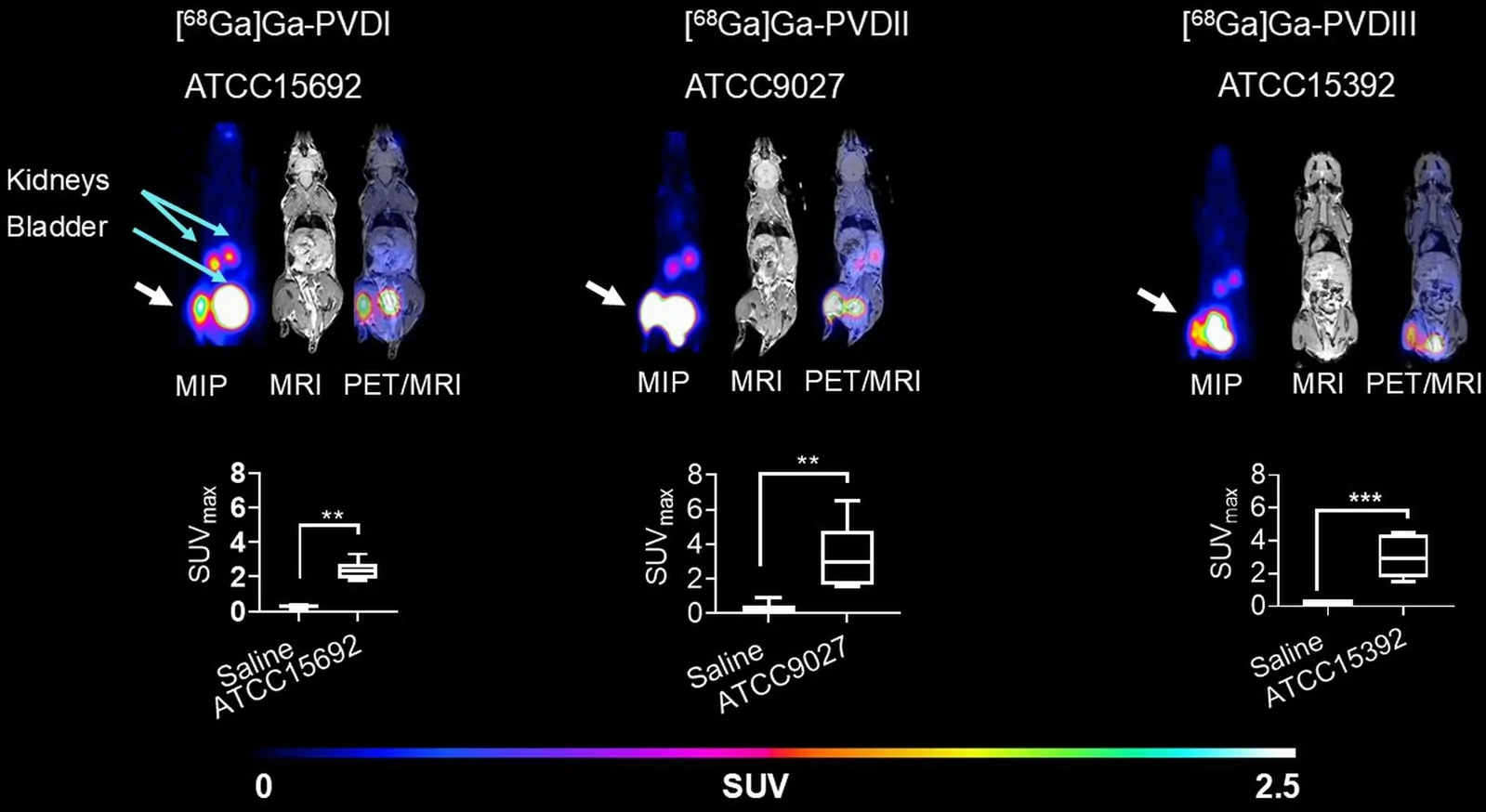
Maximum-intensity-projection (MIP), MR, and fused PET/MR sagittal slice images of mice with *P. aeruginosa* infection (strains ATCC15692, ATCC9027, ATCC15392—each representing 1 of 3 groups) in left hind leg (white arrows) and saline injection in right hind leg. All mice received 50 μL of 10^9^ viable cells/mL of bacterial suspension and were scanned 5 h after infection and 45 min postinjection. Quantification of radioactive signal uptake is shown for each image, comparing left hind legs injected with viable *P. aeruginosa* strains and right hind legs injected with saline (*n* = 3–6). **P < 0.001; ***P < 0.00001. Supplemental Figure 5 shows full combinations and quantification of each tested strain and [^68^Ga]Ga-pyoverdines.

The in vivo pathogen specificity of [^68^Ga]Ga-pyoverdines for infection caused by *P. aeruginosa* was also confirmed. The radioactive signal from [^68^Ga]Ga-PVDI, [^68^Ga]Ga-PVDII, and [^68^Ga]Ga-PVDIII clearly accumulated at the site of *P. aeruginosa* infection caused by the corresponding strain for each pyoverdine (ATCC15692, ATCC9027, and ATCC15392, respectively). *Staphylococcus aureus* infection and sterile inflammation induced in the right hind legs showed no accumulation of radioactive signal (Fig. 4). Moreover, PET/CT imaging showed focal accumulation of the radioactive signal from [^68^Ga]Ga-PVDI, [^68^Ga]Ga-PVDII, and [^68^Ga]Ga-PVDIII in the lungs of rats infected with *P. aeruginosa* strains ATCC15692, ATCC9027, and ATCC15392, respectively, 24 h after infection with 10^7^ viable bacterial cells (Fig. 5A).

**FIGURE 4.**
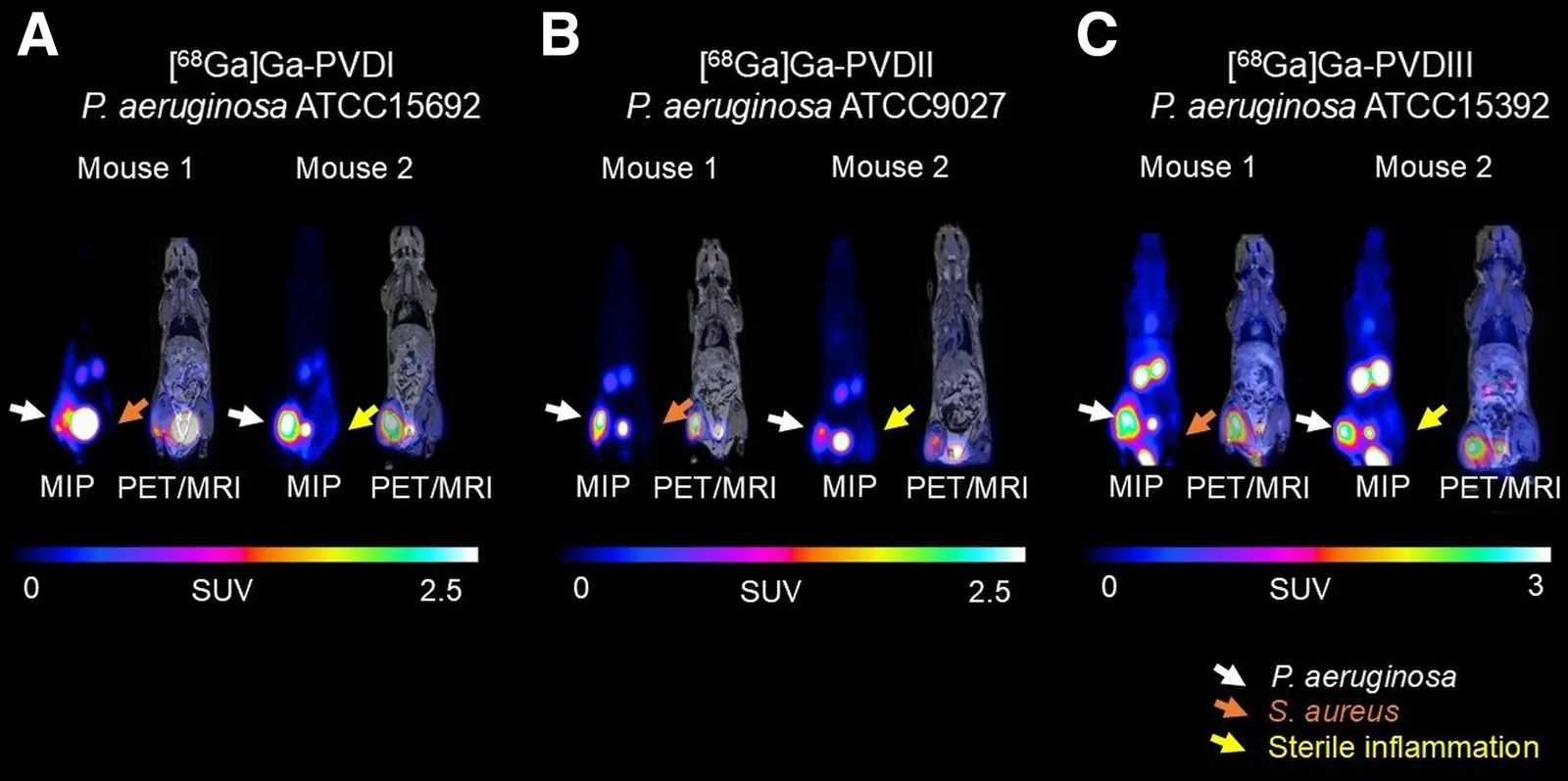
Maximum-intensity-projection (MIP) and fused PET/MR sagittal slice images with *P. aeruginosa* ATCC15692 (A) ATCC9027 (B) and ATCC15392 (C) in left hind leg (white arrows) and *S. aureus* (orange arrows) in right hind leg (mouse 1) and sterile inflammation in right hind leg (yellow arrows) (mouse 2). Mice were injected with [^68^Ga]Ga-PVDI (A), [^68^Ga]Ga-PVDII (B), and [^68^Ga]Ga-PVDIII (C) 5 h after infection (50 μL of 10^9^ viable cells/mL of *P. aeruginosa* bacterial culture and 50 μL of 10^8^ viable cells/mL of *S. aureus*). PET/MRI scans were performed 45 min postinjection.

**FIGURE 5.**
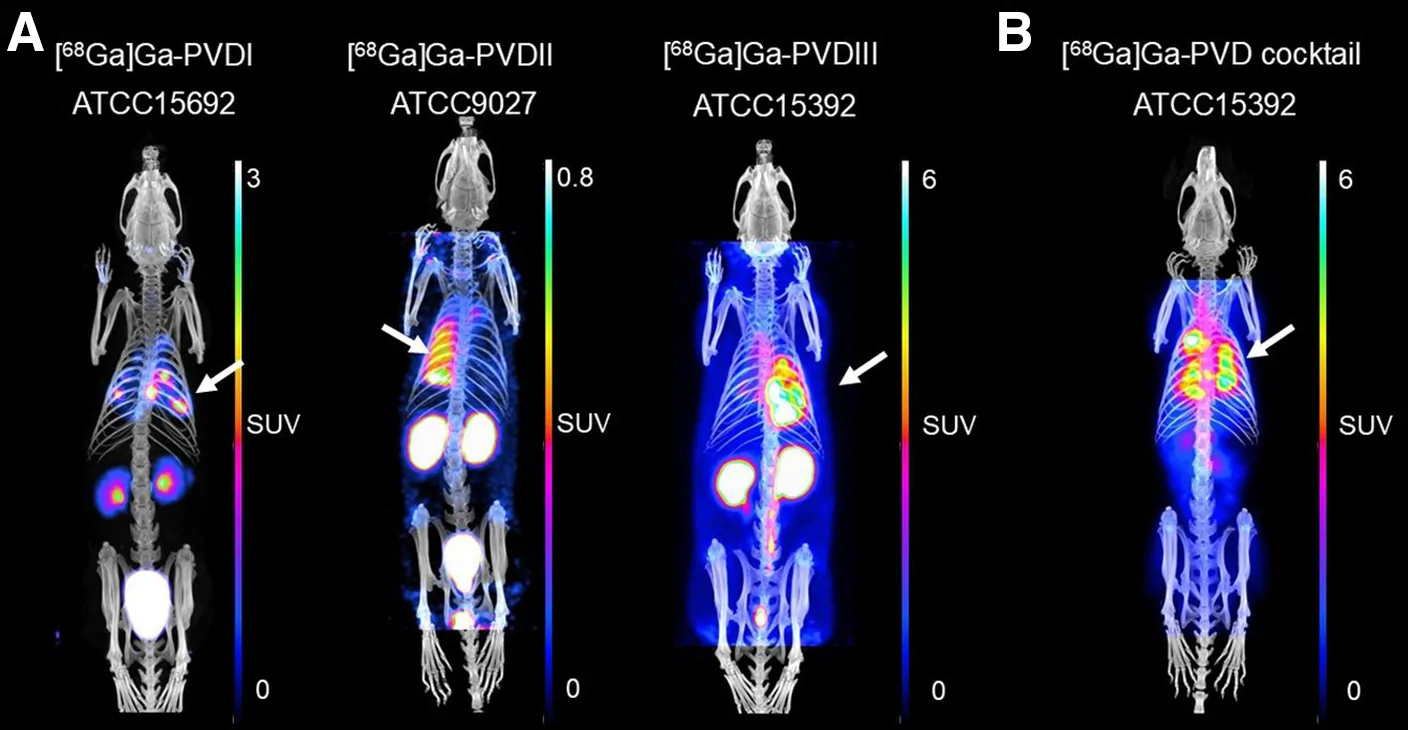
PET/CT maximum-intensity-projection images of [^68^Ga]Ga-pyoverdines in rat lung model of *P. aeruginosa* infection (24 h after infection and 45 min postinjection of [^68^Ga]Ga-PVDI (ATCC15692), [^68^Ga]Ga-PVDII (ATCC9027), [^68^Ga]Ga-PVDIII (ATCC15392) (A), and [^68^Ga]Ga-pyoverdines cocktail (ATCC15392) (b). Immunosuppressed rats received 100 μL of suspension containing (2–5) × 10^8^ viable cells/mL. White arrows indicate site of infection.

#### [^68^Ga]Ga-Pyoverdines Cocktail Imaging

The [^68^Ga]Ga-pyoverdines cocktail demonstrated specificity for imaging of *P. aeruginosa* infection. PET/MRI showed radioactive signal accumulation at the site of *P. aeruginosa* infection (strains ATCC15692, ATCC9027, and ATCC15392) in mice leg muscles. Control sites with heat-inactivated bacteria showed no accumulation of radioactivity (Fig. 6). Quantitative PET signal analysis demonstrated significant differences in the SUV_max_ between legs injected with heat-inactivated bacteria and those injected with viable bacteria (Fig. 6).

**FIGURE 6.**
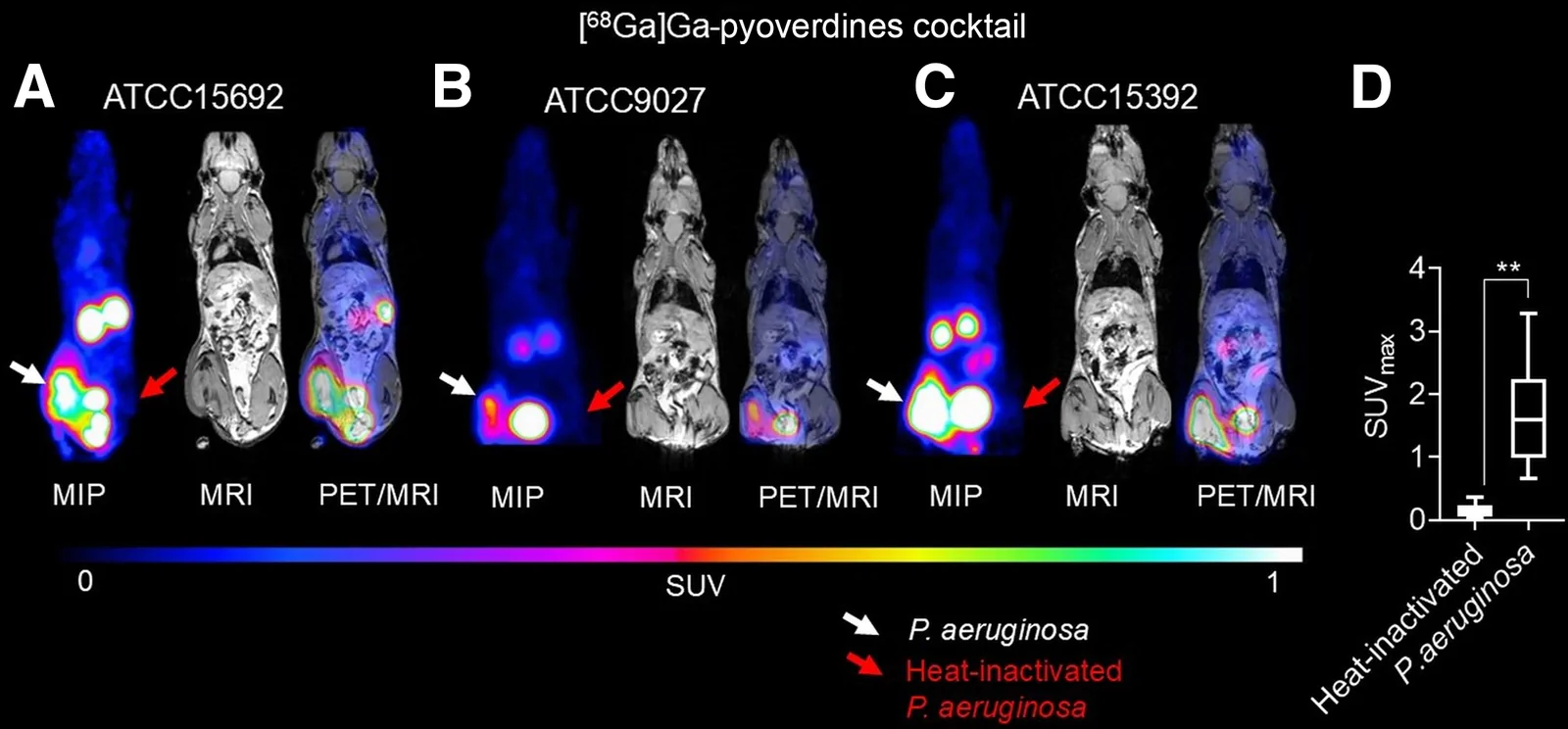
Maximum-intensity-projection (MIP) PET image, sagittal slices of MR, and fused PET/MR images of mice infected with *P. aeruginosa* ATCC15692 (A) ATCC9027 (B), and ATCC15392 (C) in left hind leg (white arrows) and corresponding heat-inactivated *P. aeruginosa* strains (red arrows) in right hind leg. All mice received 50 μL of 10^9^ viable cells/mL of bacterial suspension. Mice were injected with [^68^Ga]Ga-pyoverdines cocktail 5h after infection, and PET/MRI scans were performed 45 min postinjection. (D) Comparison of quantification of radioactive signal uptake among left hind legs injected with viable *P. aeruginosa* strains and right hind legs injected with heat-inactivated *P. aeruginosa* strains (*n* = 8). Results are expressed as SUV_max_. **P < 0.001.

PET/CT showed focal accumulation of the radioactive signal from the [^68^Ga]Ga-pyoverdines cocktail in the lungs of rats infected with *P. aeruginosa* strains ATCC15692, ATCC9027, and ATCC15392 24 h after infection with 10^7^ viable bacterial cells (Fig. 5B). Notably, the [^68^Ga]Ga-pyoverdines cocktail successfully detected lung infections, even at significantly lower infection doses (10^5^–10^6^ viable bacterial cells), and consistently identified all *P. aeruginosa* strains tested, regardless of bacterial load. In contrast, no radioactive signal was observed in the lungs of an uninfected control rat (Fig. 7). In addition, the [^68^Ga]Ga-pyoverdines cocktail allowed the progression of infection to be monitored over time, with PET/CT scans performed at 1, 2, and 3 d after infection (Supplemental Fig. 7).

**FIGURE 7.**
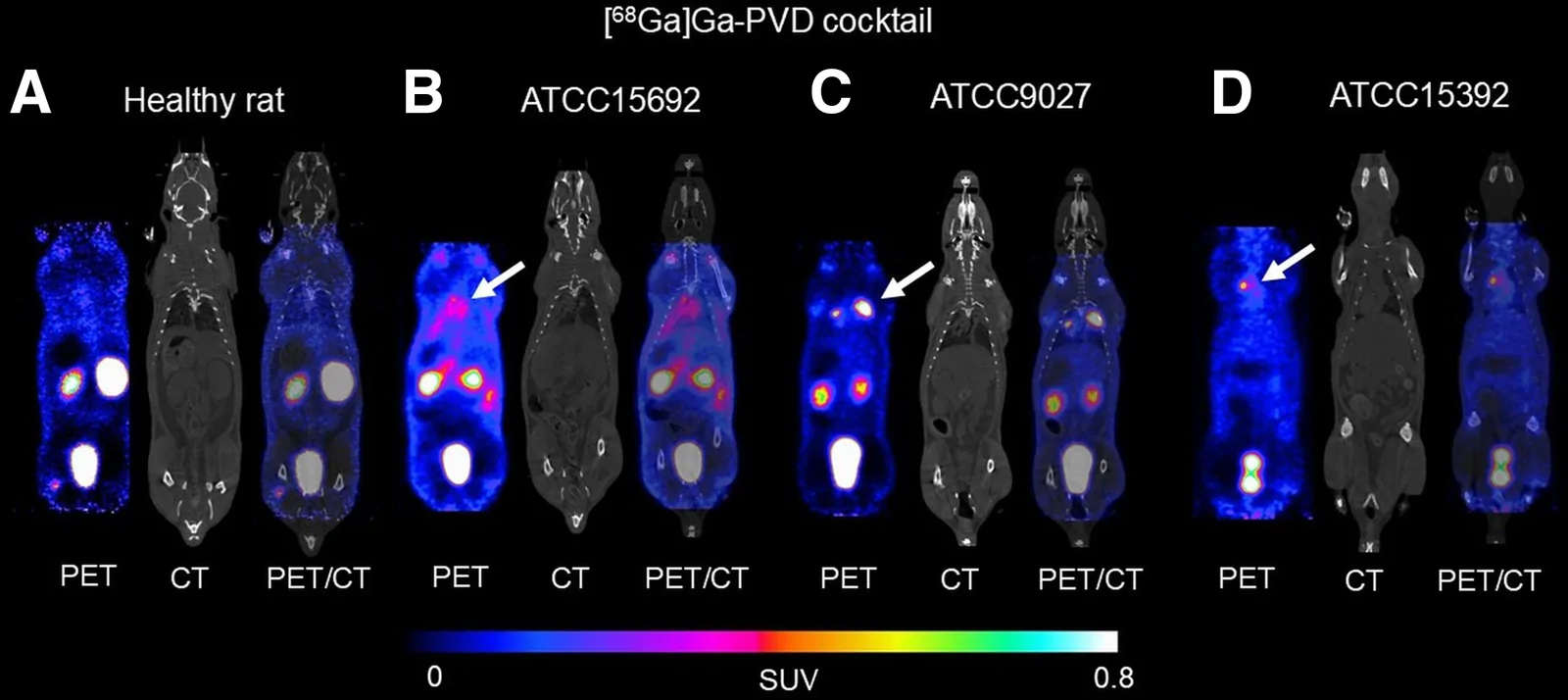
PET, CT and fused PET/CT coronal slices of images of [^68^Ga]Ga-pyoverdines cocktail in rat lung infection model. Immunosuppressed rats were infected with significantly lower infectious dose (compared with Fig. 6): 4 × 10^7^ (B), 1 × 10^7^ (C), and 6 × 10^6^ (D) viable cells/mL. Each rat received 100 μL of corresponding bacterial suspension. Imaging was performed 45 min after injection of [^68^Ga]Ga-pyoverdines cocktail, 48–72 h after infection, depending on animals’ clinical condition. Uptake shown in healthy control (A) and in lungs infected with ATCC15692 (B), ATCC9027 (C), and ATCC15392 (D). White arrows mark infection sites.

## DISCUSSION

We developed an imaging agent for the detection of a wide range of *P. aeruginosa* strains using a cocktail approach. To our knowledge, this study is the first to describe the use of a cocktail of 3 siderophore-based radiotracers for PET imaging of infection. By combining 3 [^68^Ga]Ga-pyoverdine complexes, we detected infections caused by a broad range of *P. aeruginosa* strains using PET in a mouse model of myositis and a rat model of pulmonary infection. Importantly, this tracer is highly specific for active infections, as it does not visualize nonliving bacteria, other relevant pathogens, or sterile inflammation. This offers a major advantage over ^18^F-FDG, which accumulates nonspecifically in inflammatory cells and shows high background uptake in organs such as the brain, liver, and bladder, limiting its diagnostic value (14). Although radiolabeled white blood cells offer greater specificity, their use is limited by complex handling, high cost, and suboptimal pharmacokinetics (15,16). Current research in infection imaging is focused on the development of pathogen-specific radiopharmaceuticals, such as radiolabeled antibiotics and antimicrobial peptides. More recent approaches exploit distinct aspects of bacterial biology, such as D-amino acid probes (e.g., D-[^11^C]alanine, which has shown uptake in *P. aeruginosa*), 2-deoxy-2-[^18^F]fluoro-D-sorbitol, nucleoside analogs, carbohydrates, and siderophores (4,17,18). However, many of these approaches have shown limited uptake in *P. aeruginosa*, which remains a challenging pathogen for targeted imaging of infections.

Several radiolabeled siderophores have been evaluated in preclinical studies for infection imaging, demonstrating high metabolic stability, favorable pharmacokinetics with rapid renal clearance, and specific uptake at infection sites caused by various pathogens (6,9,19–22). Among them, [^68^Ga]Ga-PVDI has previously been used for PET imaging of *P. aeruginosa* infections. However, earlier studies investigated only a single reference strain (ATCC15692) and 1 patient isolate (1019), both showing [^68^Ga]Ga-PVDI uptake (13). It is important to note that siderophore production and use are highly strain-specific across bacterial species (23). *P. aeruginosa* is highly diverse, with nearly 8000 genomes catalogued in the *Pseudomonas* genome database. To date, nearly 100 pyoverdines have been described, characterized by a conserved chromophore, an acyl side chain, and a highly variable peptide backbone (24,25). Because of these differences, *P. aeruginosa* strains are grouped into 3 siderotype classes: PVDI, II, and III (12). Building on this classification, we successfully radiolabeled all 3 pyoverdines with ^68^Ga and created a [^68^Ga]Ga-pyoverdines cocktail.

A key advantage of this approach is its ability to detect all tested *P. aeruginosa* strains, ensuring its applicability across clinical isolates and its ability to detect infection caused by relatively low infection doses. Our data in rat lung infection models with inocula of 10^5^–10^7^ viable bacterial cells established reproducible lung infections suitable for imaging studies. These bacterial burdens are within or close to the ranges typically considered clinically relevant, as diagnostic thresholds for pneumonia are in the range of 10^3^–10^6^ viable bacterial cells/mL (26). These findings therefore support the translational potential of this approach.

Biodistribution studies revealed rapid clearance of [^68^Ga]Ga-pyoverdines from the bloodstream and minimal retention in nontargeted tissues. A current limitation of the cocktail approach is the radiolabeling strategy. Currently, the cocktail is formed by combining 3 individually radiolabeled pyoverdines, which reduces the effective activity of each component, as only a fraction of the dose is relevant for a given strain. Despite this limitation, the PET signal in our in vivo studies was robust and clearly distinguishable from controls, indicating feasibility of the approach. Future improvements may include optimizing the relative composition of the cocktail or developing alternative labeling strategies to increase molar activity.

The clinical translation of radiolabeled siderophores remains challenging. Initial clinical studies using [^68^Ga]Ga-siderophores have recently been conducted with [^68^Ga]Ga-triacetylfusarinine C and [^68^Ga]Ga-DFO-B, which are currently in trials for PET imaging of fungal, bacterial, and vascular graft infections (EudraCT no. 2020-002868-31; NCT05285072; EUCT no. 2023-509744-10-00) (27). The automated production of [^68^Ga]Ga-DFO-B using cassette-based synthesis modules represents an important step toward clinical translation by simplifying preparation and reducing costs (28). The main limitation of other siderophores in terms of their clinical translation is that they rely on isolation from bacterial cultures, which is not yet widely available through Good Manufacturing Practice–compliant processes. An exception is the aforementioned DFO-B, a siderophore originally developed as an iron-chelating drug and now being repurposed as a PET tracer (20,29).

Further challenges in translation of radiolabeled siderophores into clinical practice arise when a cocktail approach is used. Although a few studies have explored radiotracer cocktails in oncology (30–32), this concept has not yet been applied to infection imaging. Translation of the cocktail approach into clinical practice would require production under Good Manufacturing Practice-compliant conditions and regulatory evaluation of the combined radiopharmaceutical (33). In principle, the individual [^68^Ga]Ga-pyoverdines could be produced using established ^68^Ge/^68^Ga generator–based radiopharmaceutical procedures and subsequently combined into a single preparation before administration. Because the radiotracers are structurally related and labeled with the same radionuclide, similar radiosynthesis and quality control procedures are expected.

The rationale for this multitracer strategy lies in the structural diversity of pyoverdines across *P. aeruginosa* strains, which may limit the sensitivity of single-ligand tracers. A cocktail approach may therefore improve detection across diverse clinical isolates. Clinically, this strategy could be particularly useful for imaging suspected *P. aeruginosa* infections in situations where pathogen identification is difficult or delayed, such as pulmonary infections in patients with cystic fibrosis and nosocomial infections in immunocompromised individuals or patients requiring mechanical ventilation (10).

Accurate and timely diagnosis is important for patients with *P. aeruginosa*, a common and potentially life-threatening pathogen, especially in immunocompromised patients. Early detection enables targeted therapy and reduces the use of broad-spectrum antibiotics, thereby helping to limit antimicrobial resistance (34). Several approaches have been explored for *P. aeruginosa*–specific radiotracers, including monoclonal antibodies, bacteriophages, and metabolic probes. Although antibodies and phages have shown good specificity, their slow clearance and poor image quality have limited clinical translation (35,36). More recently, radiotracers such as 6″-^18^F-fluoromaltotriose and ^123^I-β-methyl-*p*-iodophenylpentadecanoic acid have enabled imaging of *P. aeruginosa* (37,38). However, they lack sufficient specificity and exhibit suboptimal biodistribution.

## CONCLUSION

We successfully developed an imaging agent based on ^68^Ga-labeled pyoverdines for detecting *P. aeruginosa* infections. To our knowledge, this is the first study to use a cocktail approach combining 3 different pyoverdines to ensure detection across all *P. aeruginosa* strains tested. Uptake of [^68^Ga]Ga-pyoverdines was evaluated in several reference strains and clinical isolates, with at least one of the 3 tracers showing consistent positive uptake. Specificity was validated in vitro and confirmed in vivo using 2 animal infection models. The tracer did not accumulate in other pathogens, nonviable bacteria, or sterile inflammation, confirming its high specificity. Furthermore, the radiolabeling and imaging process is fast, providing results within 1 h. This approach represents a significant advance in accurate *P. aeruginosa* infection diagnosis, demonstrates the potential of radiolabeled siderophores for infection imaging, and lays a strong foundation for future clinical translation.

## DISCLOSURE

Financial support is provided by the National Institute of Virology and Bacteriology (EXCELES project no. LX22NPO5103), which is funded by the European Union—Next Generation EU and the Czech Ministry of Education, Youth and Sports through projects EATRIS-CZ (LM2023053), Czech-BioImaging (LM2023050), Technology Agency of the Czech Republic (TN02000109), and the Czech Science Foundation (grant 24-14579L). No other potential conflict of interest relevant to this article was reported.

## Notes

KEY POINTS **QUESTION:** Can a cocktail of [^68^Ga]Ga-pyoverdines enable targeted PET imaging *of P. aeruginosa* infections? **PERTINENT FINDINGS:** In this preclinical imaging study using rodent infection models, the [^68^Ga]Ga-pyoverdines cocktail selectively accumulated in *P. aeruginosa*–infected tissue but not in sterile inflammation or other bacterial infections, demonstrating high specificity and favorable pharmacokinetics. **IMPLICATIONS FOR PATIENT CARE:** This targeted imaging strategy offers translational potential for noninvasive diagnosis and monitoring of *P. aeruginosa* infections, particularly in complex clinical settings where rapid pathogen-specific identification is critical.

## References

[1] GBD 2021 Antimicrobial Resistance Collaborators. Global burden of bacterial antimicrobial resistance 1990–2021: a systematic analysis with forecasts to 2050. Lancet. 2024;404:1199–1226. 10.1016/S0140-6736(24)01867-1 PMC11718157 39299261

[2] GBD 2017 Causes of Death Collaborators. Global, regional, and national age-sex-specific mortality for 282 causes of death in 195 countries and territories, 1980-2017: a systematic analysis for the Global Burden of Disease Study 2017. Lancet. 2018;392:1736–1788. 10.1016/S0140-6736(18)32203-7 PMC6227606 30496103

[3] Miller JM Binnicker MJ Campbell S. A guide to utilization of the microbiology laboratory for diagnosis of infectious diseases: 2018 Update by the Infectious Diseases Society of America and the American Society for Microbiology. Clin Infect Dis. 2018;67:e1–e94. 10.1093/cid/ciy381 PMC7108105 29955859

[4] Signore A Ordonez AA Arjun C. The development and validation of radiopharmaceuticals targeting bacterial infection. J Nucl Med. 2023;64:1676–1682. 10.2967/jnumed.123.265906 PMC10626374 37770110

[5] Roll W Faust A Hermann S Schäfers M. Infection imaging: focus on new tracers? J Nucl Med. 2023;64 (suppl 2):59S–67S. 10.2967/jnumed.122.264869 37918846

[6] Petrik M Pfister J Misslinger M Decristoforo C Haas H. Siderophore-based molecular imaging of fungal and bacterial infections—current status and future perspectives. J Fungi (Basel). 2020;6:73. 10.3390/jof6020073 PMC7345832 32485852

[7] Hider RC Kong X. Chemistry and biology of siderophores. Nat Prod Rep. 2010;27:637–657. 10.1039/b906679a 20376388

[8] Petrik M Haas H Schrettl M Helbok A Blatzer M Decristoforo C. In vitro and in vivo evaluation of selected ^68^Ga-siderophores for infection imaging. Nucl Med Biol. 2012;39:361–369. 10.1016/j.nucmedbio.2011.09.012 PMC3314960 22172389

[9] Petrik M Zhai C Haas H Decristoforo C. Siderophores for molecular imaging applications. Clin Transl Imaging. 2017;5:15–27. 10.1007/s40336-016-0211-x PMC5269471 28138436

[10] Qin S Xiao W Zhou C. *Pseudomonas aeruginosa*: pathogenesis, virulence factors, antibiotic resistance, interaction with host, technology advances and emerging therapeutics. Signal Transduct Target Ther. 2022;7:199. 10.1038/s41392-022-01056-1 PMC9233671 35752612

[11] Graña-Miraglia L Geney Higuita JL Salazar JC Guaya Iñiguez D Alcolado León C García-Angulo VA. Total substitution and partial modification of the set of non-ribosomal peptide synthetases clusters lead to pyoverdine diversity in the *Pseudomonas fluorescens* complex. Front Microbiol. 2024;15:1421749. 10.3389/fmicb.2024.1421749 PMC11366639 39224222

[12] Meyer J-M Stintzi A De Vos D. Use of siderophores to type pseudomonads: the three *Pseudomonas aeruginosa* pyoverdine systems. Microbiology (Reading). 1997;143:35–43. 10.1099/00221287-143-1-35 9025276

[13] Petrik M Umlaufova E Raclavsky V. Imaging of *Pseudomonas aeruginosa* infection with Ga-68 labelled pyoverdine for positron emission tomography. Sci Rep. 2018;8:15698. 10.1038/s41598-018-33895-w PMC6200719 30356077

[14] Calabria FF Guadagnino G Cimini A Leporace M. PET/CT imaging of infectious diseases: overview of novel radiopharmaceuticals. Diagnostics (Basel). 2024;14:1043. 10.3390/diagnostics14101043 PMC11120316 38786341

[15] Signore A Lazzeri E Lauri C. Acquisition protocols and image interpretation criteria nuclear medicine imaging of infectious diseases. In: Signore A Glaudemans AWJM, eds. Nuclear Medicine In Infectious Diseases. Vol. 1. Springer International Publishing; 2020:61–71.

[16] Bhargava KK Palestro CJ. White blood cell labeling with ^18^F, ^64^Cu, and ^89^Zr. In: Signore A, ed. Nuclear Medicine And Molecular Imaging. Elsevier; 2022:305–309.

[17] Mota F Ordonez AA Firth G Ruiz-Bedoya CA Ma MT Jain SK. Radiotracer development for bacterial imaging. J Med Chem. 2020;63:1964–1977. 10.1021/acs.jmedchem.9b01623 PMC7069783 32048838

[18] Kahts M Summers B Gutta A Pilloy W Ebenhan T. Recently developed radiopharmaceuticals for bacterial infection imaging. EJNMMI Radiopharm Chem. 2024;9:49. 10.1186/s41181-024-00279-7 PMC11187059 38896373

[19] Bendova K Raclavsky V Novotny R. [^68^Ga]Ga-ornibactin for *Burkholderia cepacia complex* infection imaging using positron emission tomography. J Med Chem. 2023;66:7584–7593. 10.1021/acs.jmedchem.3c00469 PMC10258796 37252893

[20] Petrik M Umlaufova E Raclavsky V. ^68^Ga-labelled desferrioxamine-B for bacterial infection imaging. Eur J Nucl Med Mol Imaging. 2021;48:372–382. 10.1007/s00259-020-04948-y PMC7835195 32734456

[21] Krasulova K Neuzilova B Dvorakova Bendova K. Preclinical characterisation of gallium-68 labeled ferrichrome siderophore stereoisomers for PET imaging applications. EJNMMI Radiopharm Chem. 2024;9:20. 10.1186/s41181-024-00249-z PMC10912063 38436776

[22] Dvorakova Bendova K Krasulova K Neuzilova B. Positron emission tomography imaging of *Acinetobacter baumannii* infection: comparison of gallium-68 labeled siderophores. ACS Infect Dis. 2025;11:917–928. 10.1021/acsinfecdis.4c00946 PMC11997986 40099411

[23] Schalk IJ. Bacterial siderophores: diversity, uptake pathways and applications. Nat Rev Microbiol. 2025;23:24–40. 10.1038/s41579-024-01090-6 39251840

[24] Winsor GL Griffiths EJ Lo R Dhillon BK Shay JA Brinkman FSL. Enhanced annotations and features for comparing thousands of *Pseudomonas* genomes in the *Pseudomonas* genome database. Nucleic Acids Res. 2016;44:D646–D653. 10.1093/nar/gkv1227 PMC4702867 26578582

[25] Budzikiewicz H Schäfer M Fernández DU Matthijs S Cornelis P. Characterization of the chromophores of pyoverdins and related siderophores by electrospray tandem mass spectrometry. Biometals. 2007;20:135–144. 10.1007/s10534-006-9021-3 16927174

[26] Ioanas M Ferrer R Angrill J Ferrer M Torres A. Microbial investigation in ventilator-associated pneumonia. Eur Respir J. 2001;17:791–801. 10.1183/09031936.01.17407910 11401077

[27] Sim BZ Levy S Haskali M. P-2126. ‘SPECIFIC’ first-in-human clinical trial of ^68^Ga-triacetylfusarinine (TAFC) siderophore PET/CT for detection of invasive aspergillosis [abstract]. Open Forum Infect Dis. 2025;12(suppl 1): S1256.

[28] Kraihammer M Petřík M Rangger C. Automated production of [^68^Ga]Ga-desferrioxamine B on two different synthesis platforms. Pharmaceutics. 2024;16:1231. 10.3390/pharmaceutics16091231 PMC11435116 39339267

[29] Payne KA Rofail D Baladi J-F. Iron chelation therapy: Clinical effectiveness, economic burden and quality of life in patients with iron overload. Adv Ther. 2008;25:725–742. 10.1007/s12325-008-0085-z 18704280

[30] Iagaru A Mittra E Yaghoubi SS. Novel strategy for a cocktail ^18^F-fluoride and ^18^F-FDG PET/CT scan for evaluation of malignancy: results of the pilot-phase study. J Nucl Med. 2009;50:501–505. 10.2967/jnumed.108.058339 19289439

[31] Harisankar CNB Agrawal K Bhattacharya A Mittal BR. F-18 fluoro-deoxy-glucose and F-18 sodium fluoride cocktail PET/CT scan in patients with breast cancer having equivocal bone SPECT/CT. Indian J Nucl Med. 2014;29:81–86. 10.4103/0972-3919.130287 PMC3996776 24761058

[32] Simoncic U Perlman S Liu G Jeraj R. Optimizing an ^18^F-NaF and ^18^F-FDG cocktail for PET assessment of metastatic castration-resistant prostate cancer. Nucl Med Commun. 2015;36:1174–1180. 10.1097/MNM.0000000000000383 PMC4626289 26378490

[33] Huang Y-Y. An overview of PET radiopharmaceuticals in clinical use: regulatory, quality and pharmacopeia monographs of the United States and Europe. https://www.intechopen.com/chapters/62269. Published November 5, 2018. Accessed July 30, 2026.

[34] Welling MM Hensbergen AW Bunschoten A Velders AH Roestenberg M van Leeuwen FWB. An update on radiotracer development for molecular imaging of bacterial infections. Clin Transl Imaging. 2019;7:105–124.

[35] Rubin RH Young LS Hansen WP. Specific and nonspecific imaging of localized Fisher immunotype 1 *Pseudomonas aeruginosa* infection with radiolabeled monoclonal antibody. J Nucl Med. 1988;29:651–656. 3131499

[36] Elena Cardoso M Fernández L Tejería E Esperón P Terán M. Evaluation of a labelled bacteriophage with ^99m^Tc as a potential agent for infection diagnosis. Curr Radiopharm. 2016;9:137–142. 10.2174/1874471009666160215162023 26876366

[37] Gowrishankar G Hardy J Wardak M. Specific imaging of bacterial infection using 6″-^18^F-fluoromaltotriose: a second-generation PET tracer targeting the maltodextrin transporter in bacteria. J Nucl Med. 2017;58:1679–1684. 10.2967/jnumed.117.191452 PMC5632736 28490473

[38] Nishiyama Y Mizutani A Kobayashi M. SPECT imaging of *P. aeruginosa* infection in mice using ^123^I-BMIPP. Pharmaceutics. 2024;16:656. 10.3390/pharmaceutics16050656 PMC11124952 38794318

